# First Nations Approaches to Childhood Obesity: Healthy Lifestyles in Canada Compared with Alternatives for Alaska Native Communities

**DOI:** 10.3390/children4050038

**Published:** 2017-05-11

**Authors:** Peter A. de Schweinitz, Janet M. Wojcicki

**Affiliations:** 1 Family Medicine, Tanana Chiefs Conference, Fairbanks, AK 99701, USA; peter.deschweinitz@tananachiefs.org; 2 Department of Pediatrics, University of California, San Francisco, San Francisco, CA 94158, USA

**Keywords:** First Nations, Alaska Native, Alaska, rural, childhood obesity

## Abstract

Alaska Native and American Indian children have among the highest prevalence of obesity in the United States. Canadian Aboriginal populations including First Nations also have high rates of obesity but obesity rates among children are noticeably lower. We highlight some of the important differences between American and Canadian approaches to healthy lifestyles and Aboriginal/Native health, including diet and physical activity, which may in part explain the differences in obesity prevalence. Specifically, the Canadian government provides a food subsidy program to bring perishable fruits and vegetable to remote, rural Canadian areas and secondly supports the use of traditional foods and harvesting/gathering through a number of government supported programs. Lastly, there may be a better sense of community and overall life satisfaction for Aboriginals compared with Alaska Natives, in part because of the incorporation of healthcare and other services within the larger overall community, as opposed to separate services as is the case for Alaska Natives. This perspective provides insight into some of these potential differences.

## 1. Introduction

Obesity rates have increased dramatically in adults from 14.6% in the early 1970s to 35.3% in the period 2007–2010 in the United States. At present, American Indian and Alaska Native peoples are among those groups in the United States with the highest obesity rates, with obesity defined as having a body mass index (BMI) greater than or equal to 30 for adults and greater than or equal to the 95th percentile for children [1]. As of 2015, 14.1% of Alaska Native high school youth were obese [2], and in the period 2010–2014 Alaska Native adults had an obesity prevalence of 35.2% [3]. Although obesity rates vary by region, as a composite group, young Alaska Native/American Indian children report some of the highest obesity rates of any racial or ethnic group in the United States. Among Alaska Native preschool 3 year-olds, 42.2% were obese in the period 2008–2009 [4,5], and children age 5 to 12 years old had an obesity prevalence of 32.0% in 2013 [6].

In Canada, obesity prevalence among First Nations and Inuit peoples is also high, with 26% of adults being obese and 31.3% overweight (BMI between 25 and less than 30) [7]. Aboriginal populations on the two sides of the international border share similar genetic and cultural inheritance. However, Canadian Aboriginal populations have differences in lifestyle and dietary practices compared to their neighboring Alaskan Native populations. In particular, these behavioral differences impact younger Aboriginal children (6–14 years old), who have a lower obesity prevalence than Alaska Native peoples as per the Aboriginal Children’s Survey (18.8% obesity for off-reserve First Nations children versus 32% for Alaska Native persons) [6,8]. Of note, the majority of First Nations peoples live off-reserve in Canada (approximately one-third of First Nations live on-reserve) [9].

As we discuss in this commentary, we hypothesize that there is an overall healthier approach to diet and lifestyle for Canadian Aboriginal peoples as compared to Alaska Native peoples. While the data and methods we employ are insufficient to validate our hypothesis, we hope to stimulate further research and discussion in this area.

## 2. Patterns of Disease in Alaskan Populations

Historically, type 2 diabetes and obesity were rare in Alaska Native populations. However, by the mid-1950s, many Alaska Native peoples had adopted a Western lifestyle [10], specifically the sedentary habits and processed food consumption patterns of the general U.S. population. Similarly, First Nations populations had little obesity or metabolic disease until the 1950s with the introduction of Western processed foods and lifestyle choices [11].

Currently, Alaskans living in rural locations are significantly more likely to consume sugary drinks each day than Alaskans living in other regions of the state, and Alaska Native adults are nearly twice as likely (60%) as whites (32%) to consume one or more sugary drinks each day [12,13].

## 3. Lessons from Canada

A slightly lower 26% of First Nation adults were obese and 31% were overweight in the period 2007–2010 in contrast with the higher prevalence in Alaska Native peoples. However, a much lower 6.7% of youth 12 to 17 years of age were obese with 26% being overweight or obese [8]. As mentioned above, Aboriginal youth in the Aboriginal People’s Survey (2006) had a low rate of 18.8% of obesity for the total Aboriginal population (off-reserve) [14].

### 3.1. Diet and Nutrition

While Canada clearly suffers from an obesity epidemic among its Aboriginal populations, particularly the First Nations reserve population [15], there are a number of health promoting lifestyles in Canada that are associated with better overall health outcomes. First, to provide fresh fruits and vegetables and other food products to remote locations, Canada has subsidized the shipping of these healthy foods to communities since the 1980s, first under the “Food Mail Program”, and, since 2011, under the revamped program, “Nutrition North”. Specifically, the program subsidizes perishable and nutritious foods, including fruit and vegetables as well as milk and cheeses and traditional foods commercially processed in Northern communities such as caribou or Arctic char [16]. A high percentage of aboriginal children (2–5 years) eat vegetables daily (61.9% eat them at least once or twice a day) with a higher percentage (20.4%) eating them three or more times per day [17]. This contrasts with 63% of Alaska Native preschool children who eat vegetables once or twice daily but only 7% who eat them three or more times per day [3].

A 2009 evaluation by the Canadian Government estimated that the old Food Mail Program reduced food prices by about 25% on average [18]. The report suggested that the consumption of traditional foods was found to likely reduce the consumption of soda pop by almost one can per day and that the program was more cost-effective in providing nutritious food than other options studied. Meanwhile, however, First Nations and Inuit communities still consume high amounts of sweetened drinks including soda pop (0.36 servings per day) but it is possible that these programs have reduced overall consumption, although future studies need to systematically evaluate the impact of these programs [19,20].

Some of the Canadian dietary patterns may also be explained by governmental programs that support Aboriginal access to traditional foods, although there has yet to be a systematic study to analyze trends or potential impacts. Northwest, Nunavut and Yukon Territories offer a Harvest Support Program, which provides financial means to support harvesting attempts (hunting and gathering), including the purchase of small equipment. In the Northwest Territories, the “Take a Kid Trapping” Program supports local schools and Aboriginal organizations to fund traditional harvesting practices, including hunting, trapping, fishing and outdoor activities [21]. While the Supplemental Nutrition Assistance Program (SNAP)—the former “Food Stamps” program—allows eligible Alaska families to use SNAP funds to purchase specific hunting gear and equipment if communities do not have access to grocery stores or paved roads, studies suggest that these programs are rarely used in Alaska [22].

Previous studies have found that Alaska Native individuals who consume a higher diet of traditional foods have better metabolic health and lower rates of obesity [23]. Younger Alaska Native adults and adolescents consume lower amounts of traditional foods than their elders [24], with one study finding that only 7% of adolescents consumed traditional foods [25]. This contrasts with data from First Nations where 70% of children consume traditional foods and 33% consume large game meats monthly, as per the 2006 Aboriginal Children’s Survey [17]. However, other First Nations studies do indicate that younger generations have less interest and are consuming fewer traditional foods than elders [26], although Canada, in contrast with the United States, may have programs already in place to address these issues.

### 3.2. Physical Activity and Mental Health

In addition to dietary differences and support for food access in remote locations, First Nations youth and adults report higher activity levels than their counterparts in Alaska. The percentage of Alaska Native children meeting the physical activity recommendations (60 min daily) is low, at only 25% for Alaska Native high school boys, and 14% for girls [5]. By contrast, 37% of Canadian Aboriginal adults and adolescents 12 years and over defined themselves as active, with the highest percentages for those aged 12–17 years old. Only 29.9% of the non-Aboriginal population defined themselves as active [27]. The percentage of Alaska Native high school students who had three or more hours of excessive screen time (beyond school work) was 60.5% in 2013, up from 51.2% in 2007 (Alaska Native Epidemiology Center, 2008–2012), whereas among 6–14 year olds in Canada, the mean number of hours of screen time is between 2.1 and 2.2 for Aboriginal children [14].

It is possible that differences in the integration of health services partially account for discrepancies in care for indigenous peoples in the two nations. While it is beyond the scope of this paper to detail the complexities of health care systems, in the U.S., funding for Native Americans is channeled through the Indian Health Services and managed either by Indian Health Services or tribal contractors [28]. In Canada, by contrast, specific policies are most often decided at the level of the province or the territory, although on reserve and Aboriginals populations living on traditional lands are under the purview of the federal government [29,30]. In both the U.S. and Canada, strong movements toward tribal autonomy and self-governance have shifted much control of health programming to tribal entities. All Canadians are covered by the national insurance policy, and as such there is no separate health service to ensure free care for off-reserve Aboriginals, as is the case in Alaska. Métis, off-reserve First-Nations and Inuit outside of their traditional territories are the responsibility of the territorial and provincial governments without a specific designated Aboriginal health program. Specific Aboriginal programs are more at the jurisdiction of the province or territory with the federal government employing a decentralized approach [31].

In certain areas of Canada, this decentralized approach may result in better preventative medicine against obesity. In Yukon territory, for example, there is specific health legislation which recognizes the need to respect traditional healing practices, including diet [29]. At hospitals in the Yukon, every Aboriginal patient is given access to daily consumption of traditional foods through a harvesting program that uses meat hunted by tourists. Moreover, attached to the hospital is a traditional healing unit, with traditional herbs, a space for Aboriginal families to stay, and a culturally-resonant room in which families can grieve and perform rituals when their loved ones die in the hospital. A traditional “liaison” attends to the cultural needs of each Aboriginal patient throughout his or her hospital stay [31].

These differences in diet and lifestyle, and approaches to healthy living may also translate into better mental health for First Nations versus Alaska Native families. Although suicide rates among Aboriginal peoples in Canada are high (as in Alaska), according to a national survey, the aboriginal populations in Canada report a high life satisfaction (self-reported as being satisfied or very satisfied with their lives) (92.2% for Inuit; 89.1% for First Nations) with Inuit persons in particular reporting a high percentage (81.5%) of a strong or very strong sense of belonging to a community, much higher than the 65.0% for the non-aboriginal population [7,32]. Conversely, there appears to be little difference between white and Alaska Native persons in terms of life satisfaction (asked simply about overall satisfaction with life) with both reporting high overall satisfaction (answering “satisfied” or “very satisfied”) according to the Behavioral Risk Factor Surveillance System Survey [33]. While First Nations and other Aboriginal populations in Canada are not immune to the impacts of obesity and have also suffered a substantial rise in obesity since the 1990s, some of their current programmatic approaches to addressing the problem may be more successful in the long-term in creating a healthy culture.

### 3.3. Limitations

Our commentary suggests possible differences between the United States and Canada in terms of Alaska Native and Aboriginal health outcomes. We are presenting hypotheses that should be further investigated. As the studies evaluated in this paper may be using different metrics to assess lifestyle, including dietary intake and physical activity as well as self-reported BMI indicators to assess obesity and overweight, in contrast to in-person measurements, our conclusions need to be taken with a note of caution and replicated in future well-designed studies. Furthermore, studies are from different areas of Canada versus Alaska and are conducted in different time periods, with varied age groups of children. Further studies using standardized measurements are needed to make more definitive cross-border comparisons.
